# Inhibition of NADPH Oxidase-Derived Reactive Oxygen Species Decreases Expression of Inflammatory Cytokines in A549 Cells

**DOI:** 10.1007/s10753-019-01084-0

**Published:** 2019-10-14

**Authors:** Joanna Wieczfinska, Przemyslaw Sitarek, Ewa Skała, Tomasz Kowalczyk, Rafal Pawliczak

**Affiliations:** 1 Department of Immunopathology, Medical University of Lodz, 7/9 Zeligowskiego, Bldg 2, Rm 122, 90-752 Lodz, Poland; 2 Department of Biology and Pharmaceutical Botany, Medical University of Lodz, Lodz, Poland; 3 Department of Genetics and Plant Molecular Biology and Biotechnology, The University of Lodz, Lodz, Poland

**Keywords:** inflammatory cytokines, oxidative stress, apocynin

## Abstract

Various experimental models strongly support the hypothesis that airway inflammation can be caused by oxidative stress. Inflammatory airway diseases like asthma and COPD are characterized by higher levels of ROS and inflammatory cytokines. One of the sources of ROS is NADPH oxidase. Therefore, the aim of the study was to investigate influence of NADPH oxidase inhibition on the expression of IL-6, IL-8, TNF, TSLP, CD59, and PPAR-γ *in vitro.* A549 cells were incubated with apocynin in three concentrations (0.5 mg/ml, 1 mg/ml, and 3 mg/ml). Cells were trypsinized and RNA isolated after 1 h, 2 h, and 4 h of apocynin incubation at each concentration. Afterwards, reverse transcription was performed to evaluate mRNA expression using real-time PCR. The time-response and dose-response study showed that apocynin significantly influenced the relative expression of chosen genes (*IL-6*, *IL-8*, *TNF*, *PPAR-γ*, *TSLP*, and *CD59*). Apocynin decreased the mRNA expression of TNF-α at all concentrations used, and of IL-6 at concentrations of 1 and 3 mg/ml (*p* < 0.05). TSLP mRNA expression was also reduced by apocynin after 1 h and 2 h, and CD59 mRNA after 1 h, but only at the highest concentration. The expression of PPAR-γ was reduced after apocynin in the highest concentrations only (*p* < 0.05). The results might suggest that proinflammatory agents’ expression levels are strongly connected to the presence of oxidative stress generated by NADPH oxidase and this might be at least partially eliminated by anti-oxidative action. Apocynin, as an effective inhibitor of NADPH oxidase, seems to be useful in potential anti-oxidative and anti-inflammatory therapy.

## INTRODUCTION

Oxidative stress is a harmful process leading to the development of many respiratory inflammatory diseases, like asthma and chronic obstructive pulmonary disease (COPD) though airway damage. The superoxide is a precursor of other ROS and RNS. Inhibition of its generation seems to be an important therapeutic target. This can be achieved by the use of apocynin, a molecule that inhibits the activation of NADPH oxidase by blocking one of its subunits [1–5].

It has been shown that lung cells release inflammatory mediators and cytokines/chemokines, such as IL-6, IL-8, and TNF- α in response to oxidative stress [6, 7]. Levels of IL-6 have been shown to be elevated in a number of inflammatory diseases such as asthma and COPD [8–10]. Liu et al. report that NAC attenuated the release of particulate matter-induced IL-6 in mouse plasma, suggesting that ROS play a role of in IL-6 regulation [11].

It has been suggested that IL-6 is involved in the repair process responding to oxidative stress and depletion of reduced glutathione [12–14]. IL-6 has been demonstrated to have a protective effect against oxidative stress and mitochondrial dysfunction, as indicated by the increased toxicity of ROS in IL-6-deficient mice [14–16], and IL-6 has been found to be specifically induced as a response to disturbed redox status [14]. The increased concentration of serum IL-6 usually correlates with an increase in TNF-α concentration, both of which have a similar origin in the inflammatory processes [14]. TNF-α itself induces the expression of multiple airway epithelial cell genes, including those coding for such cytokines as IL-6 and IL-8 [17].

It is well documented that IL-8 production takes place in alveolar epithelial cells after oxidative stress [18, 19], and its inhibition might be connected with inflammatory clinical symptoms.

In COPD, it is possible that oxidative stress from cigarette smoke contributes towards elevated TSLP expression in BAL fluid [20, 21]. Nakamura et al. showed that CSE induced TSLP expression in the mouse lung in a manner dependent on oxidative stress and TNF-Α-α receptor I level [22]. Similar findings from mouse models have been observed in human asthmatic subjects, where higher concentrations of TSLP have been detected in the lungs, correlating with Th2-attracting chemokines and disease severity [20, 21, 23]. This phenomenon is connected with oxidative stress and antioxidants might potentially regulate it. Oxidative stress is also associated with a decrease in PPAR-γ expression. Blanquicett et al. demonstrated that oxidative stress, potentially through activation of inhibitory redox-regulated transcription factors, attenuates PPAR-γ expression and activity in vascular endothelial cells through suppression of PPAR-γ transcription [24]. Recent studies have suggested that oxidative stress modulates PPAR-γ; for example, H_2_O_2_-induced oxidative stress was found to significantly reduce PPAR-γ activity in renal tubular epithelial cells [25] and osteoblasts [24, 25].

CD59 is a membrane anchored complement regulatory protein that inhibits membrane attack complex (MAC) formation, thereby preventing complement-mediated cell lysis [26–28]. Li et al. detected high expression of CD59 in the tissues of patients with lung cancer. CD59 expression in non-small cell lung cancer tissues is much higher than in the surrounding tissue, and suggests that CD59 might be a biomarker for lung cancer progression [29]. Many of the known inflammatory target proteins, such as matrix metalloproteinase-9 (MMP-9), intercellular adhesion molecule-1 (ICAM-1), vascular cell adhesion molecule-1 (VCAM-1), cyclooxygenase-2 (COX-2), and cytosolic phospholipase A2 (cPLA2), are associated with NADPH oxidase activation and ROS overproduction in response to proinflammatory mediators [6, 30–35]. Thus, oxidative stress regulates both key inflammatory signal transduction pathways and target proteins involved in airway and lung inflammation [6].

Because oxidative stress, also generated by NADPH oxidase, contributes to inflammatory pathology, the aim of the study was to evaluate the influence of apocynin (NADPH oxidase inhibitor) on the expression of selected genes involved in the inflammation and antioxidant reactions in A549 cells (IL-6, IL-8, TNF-α, PPAR-γ, TSLP, and CD59).

## MATERIALS AND METHODS

### Cell Culture

A549 cells, a human adenocarcinoma cell line, were obtained from ECACC (European Collection of Cell Cultures, Heath Protection Agency, Salisbury, UK) and were grown in Ham’s F-12K medium (Sigma-Aldrich, St. Louis, MO) with 10% fetal bovine serum and 2 mM of l-glutamine (Sigma-Aldrich, St. Louis, MO). All experiments were performed after six to nine passages (*n* = 6), when the cell sheets were 80 to 90% confluent.

### Experimental Procedure

A549 cells were incubated with apocynin at three concentrations (0.5 mg/ml, 1 mg/ml, and 3 mg/ml). These concentrations were selected experimentally as the most effective. The experiments were also performed in the absence of the stimulus (control).

The cells were trypsinized and RNA was isolated after 1 h, 2 h, and 4 h of apocynin incubation with each concentration. Following this, reverse transcription was performed to assess mRNA expression using real-time PCR. A time-response and dose-response study showed that apocynin significantly influenced the relative expression of the selected genes (*IL-6*, *IL-8*, *TNF-Α*, *PPAR-γ*, *TSLP*, and *CD59*).

### RNA Extraction and cDNA Synthesis

Total RNA was isolated from the stimulated cells using RNeasy Cell Mini Kit with QIAshredder (Qiagen). RNA was DNase treated, purified, eluted in 30 μl of RNase-free water, and stored at − 80 °C for further analysis. Total RNA (1 μg) was reverse transcribed using High Capacity cDNA kit (Applied Biosystems, Foster City, CA, USA). All procedures were carried out according to the protocols given by the producer.

### Analysis of Gene Expression

Real-time PCR was conducted in order to indicate the changes in expression of *IL-6*, *IL-8*, *TNF-Α*, *PPAR-γ*, *TSLP*, and *CD59*. cDNA was subjected to qPCR using the assays designed for the selected genes (Life Technologies, Carlsbad, CA); each sample was measured in duplicate using a TaqMan analyzer 7900 (Life Technologies, Carlsbad, CA). Using the 2^−ΔΔCt^ method, data was presented as gene expression normalized to *β-actin* as an endogenous reference gene and relative to a control. The fold change of mRNA expression in each patient was calculated by comparing RQ (2^−ΔΔCt^).

### Statistical Analyses

The results were analyzed using Statistica software (v. 10.0; StatSoft, Tulsa, OK). The distribution of data and the equality of variances were checked by the Shapiro-Wilk test and Levene’s test, respectively. Significant changes were determined by ANOVA, with the appropriate *post hoc* tests as multiple comparison procedure. Values of *p <* 0.05 were considered statistically significant.

## RESULTS

### Apocynin Decreased mRNA Expression of Proinflammatory Cytokines

Lung epithelial cells in the respiratory tract are the first barrier in contact inhaled oxidants, and A549 human lung epithelial cells have been wildly used as *in vitro* models to assess airway inflammation, asthma, and respiratory sensitization, and, in case-control studies, for *in vivo* validation [36–40].

**Fig. 1:**
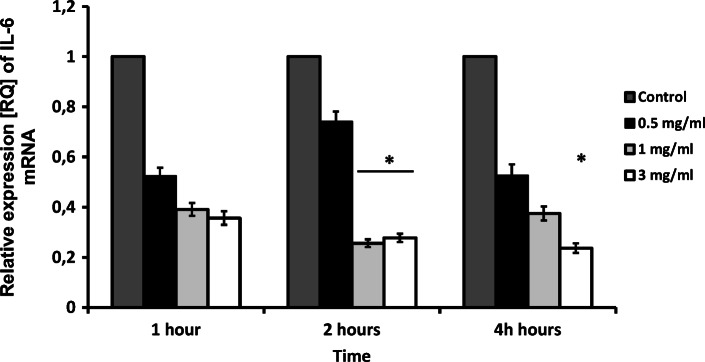
mRNA expression levels of *IL-6* in response to apocynin. Apocynin decreased IL-6 mRNA expression in A549 cells after 2 and 4 h of incubation at 1 mg/ml and 3 mg/ml concentrations (*p* < 0.05). Data presented as relative expression (RQ) ± SD, *p < 0.05.

**Fig. 2:**
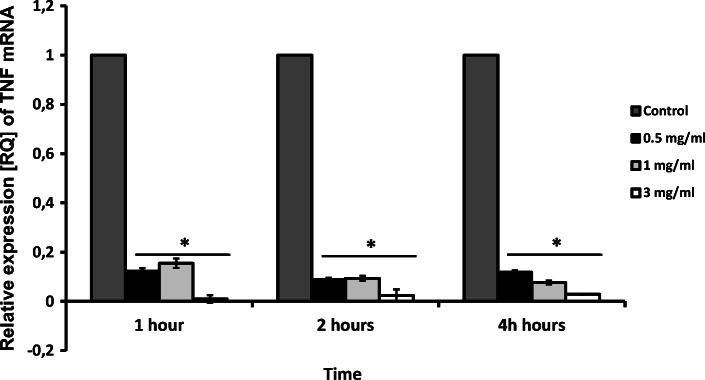
The effect of apocynin on relative expression of TNF-α in A549 cells. A549 cells presented significantly decreased expression in response to apocynin after incubation at all concentrations and in all time points (*p* < 0.05). Data presented as relative expression (RQ) ± SD, *p < 0.05.

**Fig. 3:**
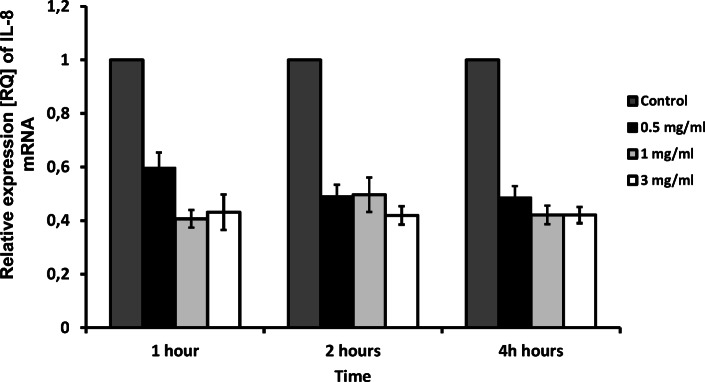
Relative expression of IL-8 after apocynin stimulation. No statistical significance in expression of IL-8 was observed after apocynin treatment (*p* > 0.05). Data presented as relative expression (RQ) ± SD.

The expression of the proinflammatory cytokines analyzed in the study appeared to be decreased by apocynin. Real-time PCR analysis revealed a significant decrease in IL-6 mRNA expression 2 h and 4 h for 3 mg/ml apocynin (*p* < 0.05), but only after 2 h for 1 mg/ml apocynin (*p* < 0.05, Fig. 1). Interestingly, apocynin significantly decreased TNF-Α mRNA expression in all doses and at all time points (*p* < 0.05, Fig. 2). However, while no significant changes in IL-8 expression were caused by apocynin, decreases in IL-8 expression were observed for all administered concentrations (*p* > 0.05, Fig. 3).

### The Effect of Apocynin on CD59 and TSLP mRNA Expression

**Fig. 4:**
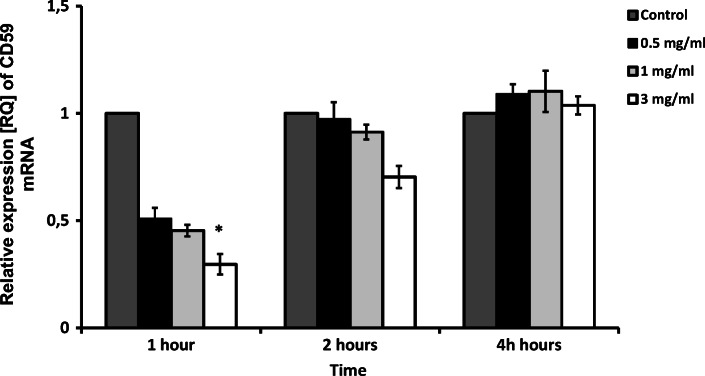
CD-59 expression in PBMC of smokers and nonsmokers. Apocynin caused significant decrease of CD-59 mRNA expression after 1 h of application at 3 mg/ml concentration only (*p* < 0.05). Data presented as relative expression (RQ) ± SD, *p < 0.05.

CD-59 mRNA expression was significantly influenced by 3 mg/ml apocynin after 1 h of application (*p* < 0.05). A similar, but insignificant, relationship was also observed after 2 h, but no such relationship was found after 4 h (*p* > 0.05, Fig. 4).

**Fig. 5:**
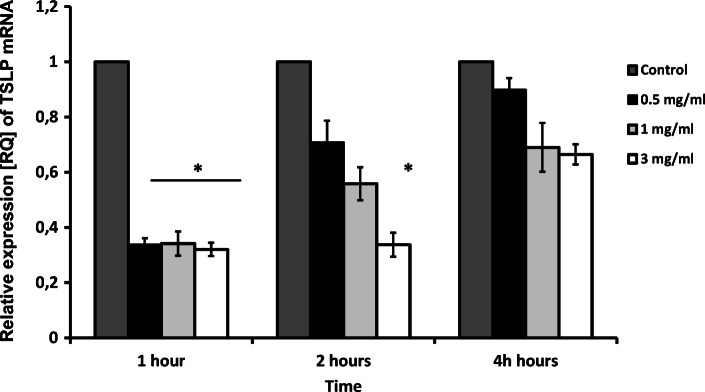
mRNA expression levels of *TSLP* in response to apocynin. Apocynin decreased TSLP mRNA expression in A549 cells after 1 h of incubation at all concentrations used and after 2 h at the highest concentrations only (*p* < 0.05). Data presented as relative expression (RQ) ± SD, *p < 0.05.

Similarly, a significant decrease of TSLP mRNA expression was observed for all doses of apocynin after 1 h of application. Only the highest concentration of apocynin significantly decreased TSLP expression after 2 h of application (*p* < 0.05), and no significant changes were observed after 4 h (*p* > 0.05); nevertheless, the trend was maintained (Fig. 5).

### Apocynin Upregulated PPAR-γ mRNA Expression

**Fig. 6:**
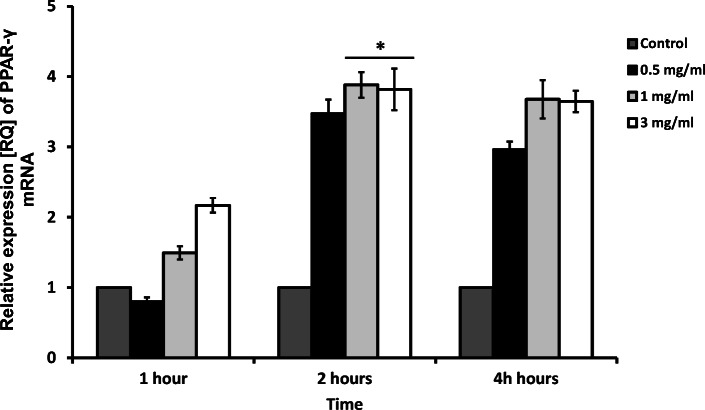
The effect of apocynin on relative expression of PPAR-γ in A549 cells. A549 cells showed an increase of PPAR-**γ** relative expression after 4 h of incubation in the two highest concentrations of apocynin (1 mg/ml and 3 mg/ml, *p* < 0.05). Data presented as relative expression (RQ) ± SD, *p < 0.05.

At each time point, apocynin increased the expression of PPAR-γ in a dose-dependent manner; however, only after 2 h of apocynin application, this change was significant (1 mg/ml and 3 mg/ml, *p* < 0.05, Fig. 6).

## DISCUSSION

As oxidative stress has previously been confirmed to play a role in the pathogenesis of many airway diseases [41–46], the present study evaluates the biological effect of the inhibition of NADPH oxidase by apocynin. To achieve this, qPCR was used to analyze the mRNA expression of selected genes, thus confirming their activity and the significance of apocynin in their potential regulation.

Our previous study on the effectiveness of inhaled apocynin on hydrogen peroxide, nitrate, and nitrite concentrations in humans found noticeable effects in healthy subjects, asthmatics, and COPD patients without any adverse effects [47–49]. In this study, unstimulated epithelial A549 cell was used. Our goal was to investigate the effect of the ROS restriction in such cells, as they might be regarded as more nearly reflecting the *in vivo* situation. ROS reduction by apocynin turned out to trigger the effect on the basal state of the cells. This might indicate further direction of the research to understand the mechanisms of this phenomenon and its consequences.

Although elevated IL-6 has long been considered a general marker of inflammation, Naik et al. suggest that IL-6 is not just a proinflammatory marker, but a key factor that contributes to the pathogenesis of some important inflammatory diseases, including asthma; it may hence serve as both a biomarker and a therapeutic target for asthma [10, 50]. Plasma levels of IL-6 are known to be increased in patients with stable COPD compared to controls [51], remain elevated for a period, and may contribute to the increased risk of depression and mortality associated with COPD [52–54].

The present study examined the expression of proinflammatory cytokines known to be associated with inflammatory airway diseases such as asthma and COPD (IL-6, IL-8, and TNF-α mRNA) after apocynin treatment. Apocynin appeared to lower the mRNA expression of each; however, only in the case of IL-6 and TNF-α were the results significant. These data confirm those of Kim et al. [55] who found that TNF-α production was significantly attenuated after treatment with apocynin in a mouse model. They also demonstrated that treatment of OVA-induced asthma mice with apocynin effectively attenuated airway lung inflammation, Th2 cytokine production, and the infiltration of inflammatory cells, such as macrophages and eosinophils, into lung tissues [55].

Kilic et al. report that application of apocynin reversed elevated levels of IL-8 [56]. These findings confirm those of Perng et al. [57] and those of our present study. Moreover, Higai et al. indicate that apocynin, next to NAC, suppressed IL-8 mRNA expression induced by glycated human serum albumin [58], which also correlates with our research.

The inhibitory effect of apocynin on the production of proinflammatory cytokines was previously demonstrated in ventilator-induced lung injury models, where treatment with apocynin repaired the structural lung injury [56, 59]. Interestingly, our findings indicate that another proiinflammatory cytokine, TSLP, was inhibited by all administered concentrations of apocynin after 1 h of application. In human bronchial epithelial cells, TSLP expression is associated with asthma severity [20, 60–62].

TSLP expression in the airway epithelium is inducible through an NF-κB-dependent pathway [54, 63, 64]. An increased number of cells expressing TSLP mRNA has been reported in the bronchi of stable COPD patients and smoking controls with normal lung function, and increased TSLP immunostaining has been shown in the smooth muscle of patients with stable COPD compared to nonsmoking subjects [21, 54, 65]. Furthermore, NF-κB, which regulates the release of many cytokines and chemokines, is shown to be responsive to oxidative stress [20, 66]. It has been proposed recently [67] that the elevated TSLP production in the bronchial mucosa in COPD may be associated with the activation of NF-κB by oxidative stress from cigarette smoke [20, 64, 68].

The expression of TSLP mRNA in neutrophils and epithelial macrophages is also significantly higher in asthmatics than healthy controls. There is also a negative correlation between levels of lung function in asthmatics and TSLP expression [21, 69]. Therefore, our results indicate oxidative stress influences TSLP level and may act as an inhibitor.

Huang et al. report that PPAR-γ activation attenuates TNF-α-enhanced ICAM-1 expression [70]. These data confirm our present results, which note that an increase of PPAR-γ mRNA expression is accompanied by a decrease of TNF-α after apocynin treatment. Previous studies have suggested that PPAR-γ activation alleviates asthmatic features, as evidenced by decreased expression of cytokines, reduced bronchoconstriction, and impaired eosinophil accumulation [71–73].

Xu et al. note that the administration of rosiglitazone, a PPAR-γ agonist, attenuated asthmatic features including airway eosinophil accumulation, inflammatory cytokine levels, smooth muscle layer thickness, and collagen deposition in a mouse model. These findings suggest that the therapeutic effect against asthma exerted by rosiglitazone was associated with activation of PPAR-γ and its downstream pathways [71].

In addition, Soletti et al. report that pharmacological PPAR-γ activation protects from smoke-induced inflammation *in vivo* in mice, and attenuates the cellular and molecular intermediates of emphysema pathogenesis in humans. *Pparg* induction in epithelial cells appears to represent a protective mechanism against cigarette smoke-induced injury response, where it may function to suppress NF-κB-mediated proinflammatory chemokine expression in an activation-dependent fashion [74].

The current study indicates that inhibition of NADPH oxidase decreased CD59 mRNA expression after 1 h. The functions of the CD59 protein are mainly involved in the MAC of human complement [75] signal stimulant, inducing the activation of T lymphocytes [76], and acting as a ligand of CD2. As bronchial epithelial cells express high levels of CD59 and that CD59 release is associated with cellular damage [28, 77], Budding et al. hypothesize that CD59 may be a marker for inflammatory lung tissue damage [26]. Therefore, the CD59-decreasing function of apocynin may shed new light on inflammation resolution and maintenance.

Apocynin appears to be a noteworthy molecule, with various promising features that can be employed in anti-oxidative and anti-inflammatory therapy; however, our study has some limitations. One such weakness is that it examines mRNA expression, but not protein expression. In addition, the A549 line used in the study are cancer cells: The main disadvantage of using cell lines is that the phenotype they express may not be consistent with the true phenotype of their primary counterparts [78]. Nevertheless, A549 cells constitute a useful *in vitro* model for studying human respiratory epithelial cell biology, as they exhibit characteristics similar to human alveolar type II cells [79].

We are aware that the weakness of the study is the lack of protein expression results and that mRNAs are not equal with regard to translation into proteins; thus, our results must be interpreted with caution. The mRNA expression alone analysis is incomplete, but shows that apocynin causes visible changes that can also be expressed at the protein level. This, however, indicates apocynin as a molecule worthy of interest.

With awareness of the effects of ROS on airway inflammation growing, antioxidant interventions have become a popular therapeutic target. Different research teams have reported that α-tocopherol and vitamin C combination therapy was effective in mitigating the effect of ozone-induced lung function decrements in asthmatics [80–83], or in normal volunteers who had consumed an antioxidant-depleted diet for 3 weeks to mimic a state of poor antioxidant nutritional status [81, 83].

In conclusion, oxidative stress plays an important role in many diseases, especially in inflammatory diseases. Its strong and effective inhibition might help to reduce inflammation and local pathogenic changes. The results presented in the current study suggest that inhibition of NADPH oxidase might be a potential target in inflammatory diseases, and apocynin seems to be an interesting molecule in this regard; however, wider studies are needed to specifically explore this topic.
